# Advancing women’s, children’s, and adolescents’ health and equity

**DOI:** 10.1136/bmj.l7010

**Published:** 2020-01-27

**Authors:** Nicholas K Alipui, Elizabeth Mason

**Affiliations:** 1 New Rochelle, New York, USA; 2 London, UK

## Abstract

Stronger accountability is key

In 2015 the UN secretary general launched the Every Woman Every Child (EWEC) global strategy to give new momentum to the urgent task of transforming the health of women, children, and adolescents by 2030.^1^ One of its recommendations was to convene an independent panel to regularly review progress on the global strategy’s targets and the sustainable development goals (SDGs).

The EWEC independent accountability panel was established in 2016 and advocates for a shared understanding of accountability founded on human rights. The panel uses a monitor, review, remedy, and act framework that has evolved over a decade of experience.^2^ Our role is to review the results of progress reports on the health of women, children, and adolescents and to make recommendations to tackle failings

A new collection of articles published by *The BMJ* and *BMJ Global Health* (www.bmj.com/leaving-no-one-behind) offers insight into how countries are progressing towards their EWEC and SDG targets with a focus on equity. Two overarching issues that could prevent countries achieving their targets by 2030 are apparent.

Firstly, as noted in several articles in the collection, global progress is too slow and too uneven. Barriers to progress include violation of rights and structural disparities in society, such as entrenched gender and socioeconomic inequalities and failure of health services to reach those most in need, especially in countries afflicted by conflict.

Secondly, accountability mechanisms and culture are weak in many countries and organisations. They often fail, for example, to uncover and redress corruption, a proved cause of health inequity.^3^ To achieve the global strategy targets and promote equity in health and sustainable development, we contend that accountability urgently requires strengthening at all levels.

## Who is accountable?

Governments have primary responsibility for achieving health and sustainable development goals and for upholding human rights. They are also responsible for using inclusive, transparent accountability mechanisms to identify failings and drive improvements.

Accountability should be ingrained in the culture of governments as the foundation for every decision and policy. The same should apply to businesses, organisations, and all stakeholders. Emerging evidence shows that when accountability mechanisms are in place, people are more likely to undertake actions to achieve goals.^4^ ^5^

## Strengthen rights based accountability

Rights based accountability is needed to effectively redress health inequities and deep seated structural disparities.^4^ ^6^ For example, research shows that in the US, black women are three to four times more likely to die from pregnancy related causes than white women.^7^ In Nigeria, inequities are seen across ethnic and tribal differentials—for example, through significant disparities in mortality in children under 5 years.^8^

Rights based accountability can explicitly redress systemic disparities, inequalities, and violations of rights such as these since it is founded in national and international law and requires translation to policies, institutions, and procedures. The universal periodic review^9^ of the UN Human Rights Council can help countries to review and remedy rights violations and structural disparities that affect health equity.

## Monitoring inequities

Global data and health estimates show which regions and countries are furthest behind in implementing the EWEC global strategy, notably countries in sub-Saharan Africa and South Asia. However, country level data have many gaps because of inadequate or non-existent monitoring systems. This is a failure of accountability and a major barrier to identifying populations whose rights are violated by lack of access to essential health services. Countries urgently need support and investment to generate high quality health and demographic data to inform decisions.

Whenever possible, data should be available in real time to facilitate rapid analysis and inform effective action that can remedy inequities and rights violations. Making data available that are disaggregated by income, gender, age, race, ethnicity, and other important factors (as recommended by SDG target 17.18)^10^ is essential to identify people in greatest need.

Countries cannot do this work alone. Our panel requests that UN agencies and other stakeholders increase their support to countries to monitor implementation of the EWEC global strategy, with particular focus on human rights and health equity. Expert partners and equity related tools, as noted in this collection, can also support countries in this regard.

## Ensuring inclusive sociopolitical participation

Stronger data systems are important but are insufficient to ensure full accountability. Inclusive participation from all stakeholders is needed to highlight barriers to progress that might not show up in regular monitoring data, such as abusive or corrupt practices in health systems. Sociopolitical accountability centres on “custodians of accountability” in every country, such as parliaments, civil society, media, communities, and individuals. Support is available to help countries ensure and institutionalise inclusive sociopolitical participation and accountability. For example, the UN, Inter-Parliamentary Union, UHC2030, the Partnership for Maternal, Newborn and Child Health, the Global Fund, and others are active in this area. We also recommend that countries track sociopolitical accountability indicators—for example, budget transparency, corruption, media freedom, citizens’ voices, and inclusive participation.

Stronger accountability is essential for women’s, children’s, and adolescents’ health. We call on countries to take the lead, with the support of the global community, to strengthen rights based accountability, monitor inequities, and ensure inclusive sociopolitical participation in order to keep the global promise that no one will be left behind in the SDGs.^10^

## Notes

Provenance and peer review: Commissioned; not externally peer reviewed.

This article is part of a series proposed by Countdown to 2030 for Women’s, Children’s and Adolescents’ Health and the Partnership for Maternal, Newborn and Child Health (PMNCH) hosted by the World Health Organization and commissioned by *The BMJ*, which peer reviewed, edited, and made the decisions to publish these articles. Open access fees are funded by the Bill and Melinda Gates Foundation and PMNCH.
